# ***In Vitro*** Assessment of Uptake and Lysosomal Sequestration of Respiratory Drugs in Alveolar Macrophage Cell Line NR8383

**DOI:** 10.1007/s11095-015-1753-8

**Published:** 2015-07-30

**Authors:** Ayşe Ufuk, Graham Somers, J. Brian Houston, Aleksandra Galetin

**Affiliations:** Centre for Applied Pharmacokinetic Research, Manchester Pharmacy School, The University of Manchester, Stopford Building, Oxford Road, Manchester, M13 9PT UK; GlaxoSmithKline, Medicines Research Centre, Stevenage, UK

**Keywords:** alveolar macrophages, lysosomal sequestration, NR8383, respiratory drugs

## Abstract

**Purpose:**

To assess accumulation and lysosomal sequestration of 9 drugs used in respiratory indications (plus imipramine as positive control) in the alveolar macrophage (AM) cell line NR8383.

**Methods:**

For all drugs, uptake at 5 μM was investigated at 37 and 4°C to delineate active uptake and passive diffusion processes. Accumulation of basic clarithromycin, formoterol and imipramine was also assessed over 0.1–100 μM concentration range. Lysosomal sequestration was investigated using ammonium chloride (NH_4_Cl), monensin and nigericin. Impact of lysosomal sequestration on clarithromycin accumulation kinetics was investigated.

**Results:**

Both cell-to-medium concentration ratio (K_p_) and uptake clearance (CL_uptake_) ranged > 400-fold for the drugs investigated. The greatest K_p_ was observed for imipramine (391) and clarithromycin (82), in contrast to no accumulation seen for terbutaline. A concentration-dependent accumulation was evident for the basic drugs investigated. Imipramine and clarithromycin K_p_ and CL_uptake_ were reduced by 59–85% in the presence of NH_4_Cl and monensin/nigericin, indicating lysosomal accumulation, whereas lysosomal sequestration was not pronounced for the other 8 respiratory drugs. Clarithromycin uptake rate was altered by NH_4_Cl, highlighting the impact of subcellular distribution on accumulation kinetics.

**Conclusions:**

This study provides novel evidence of the utility of NR8383 for investigating accumulation and lysosomal sequestration of respiratory drugs in AMs.

**Electronic supplementary material:**

The online version of this article (doi:10.1007/s11095-015-1753-8) contains supplementary material, which is available to authorized users.

## Introduction

Understanding drug disposition in the lungs remains a challenging task in the development of new medications for asthma, chronic obstructive pulmonary disease and bacterial infections (1). Several factors may affect the therapeutic efficacy of inhaled drugs including delivery devices, formulation and dose, drug physicochemical properties, metabolism and lung clearance mechanisms and disease state (2–4). Phagocytic alveolar macrophages (AMs) represent important components of clearance mechanism of drugs from the respiratory airways. While accumulation of certain drugs in AMs is required for their therapeutic effect (*e.g.*, macrolide antibiotics), this process could affect the efficacy and duration of drugs targeting submucosal compartments such as β_2_-agonists and antimuscarinics. In addition, pronounced accumulation of basic drugs (often cationic amphiphilic drugs, *i.e.*, CADs) in lysosomes of AMs may lead to drug induced phospholipidosis and the presence of foamy macrophages in the airways (5). Lysosomal sequestration of CADs has been attributed to the pH differences between cytosol (pH = 7.2) and lysosomes, as the most acidic cell organelles (pH = 4.7–4.8) (6). The unionised form of basic drugs can rapidly diffuse into lysosomes where they become predominantly ionised and ‘trapped’ as a result of the limited permeability of lysosomal membrane to ionised drugs. Lysosomal sequestration may lead to significantly higher intracellular drug concentrations which may have important therapeutic or toxicological consequences (7,8). In addition, drug-drug interactions involving lysosomotropic drugs have been proposed as an important implication of lysosomal drug sequestration (8,9). *In vitro* methods employed to investigate lysosomal sequestration include both direct approaches such as measuring drug concentrations in isolated lysosomes following homogenisation (10–12) and indirect methods based on the use of chemical agents to abolish lysosome-cytosol pH gradient (*e.g.*, ammonium chloride (NH_4_Cl), monensin and nigericin) (12–14). Reduction in drug accumulation in the presence of these indirect agents relative to control provides the extent of lysosomal accumulation (reference studies provided in the Supplementary Material, Table S1). In addition, assays involving the use of lysosome specific fluorescent dye, LysoTracker Red (LTR), have also been reported for semiquantitative investigation of lysosomal drug sequestration (14–16).

NR8383 is an AM cell line originally derived by bronchoalveolar lavage procedure of an adult male Sprague-Dawley rat; this cell line was shown to exhibit several characteristics and functions of freshly isolated rat AMs (17,18). Previous uptake studies in NR8383 focused on a single drug per study and mainly on antibiotics used for respiratory infections (19,20). To date, there are no studies investigating the accumulation in AMs of a substantial number of respiratory drugs targeting other disease areas including asthma and chronic obstructive pulmonary disease.

The aim of this study was to systematically assess the extent of intracellular accumulation in AMs and the contribution of lysosomal sequestration for nine selected drugs. All drugs in the dataset are designed or used in respiratory indications, cover a wide range of physicochemical properties and have limited *in vitro* accumulation data reported in AMs. The selected drugs and their respective physicochemical properties are summarised in Table I, together with the location of the drug target (intra- or extracellular). The corresponding chemical structures are shown in the Supplementary Material Figure S1. Drug uptake into AMs was investigated at a single substrate concentration at 37°C and 4°C to determine the dependence of cellular accumulation on active uptake and passive diffusion processes, respectively. In addition, the accumulation of a number of selected drugs in AMs was investigated over a range of substrate concentrations. In order to assess its contribution to uptake into AMs, lysosomal sequestration of the drugs was investigated in the presence of agents that abolish lysosome-cytosol pH gradient; lysosomotropic drug imipramine was included as a positive control in the studies. Cell-to-medium concentration ratio (K_p_) was determined in the presence and absence of these agents to evaluate the extent of lysosomal accumulation. The *in vitro* data were supported by staining of the cells with LTR under control conditions and in the presence of a number of basic drugs and the above mentioned chemical agents. Recommendations of the application of NR8383 for the assessment of accumulation and lysosomal sequestration of respiratory drugs are provided.

**Table I:** Physicochemical Properties of 10 Drugs Selected for the Investigation of Drug Uptake and Lysosomal Sequestration in NR8383 Cell Line

Drug	Target	LogP	LogD_7.4_	pK_a_a_	pK_a_b_	PSA (Å^2^) ^a^	Acid–base Property	References
Clarithromycin ^c^	Intracellular bacteria	3.16	1.57	–	8.99	183	Base	(21)
Imipramine	Extracellular serotonin receptor	4.80	2.38	–	9.50	6.50	Base	(22–24)
Formoterol ^d, e^	Extracellular β_2_-receptor	1.99 ^a^	2.60	10.1, 11.8 ^a^	8.14 ^a^	90.8	Base	(25)
Fenoterol ^d^	Extracellular β_2_-receptor	1.09 ^a^	0.74	9.40, 10.1 ^a^	8.25 ^a^	93.0	Base	(25)
Terbutaline ^d^	Extracellular β_2_-receptor	0.90	−1.50 ^b^	8.60, 11.0	9.90	72.7	Base	(26–28)
Budesonide ^d^	Extracellular glucocorticoid receptor	2.47 ^a^	2.47 ^a^	n/a		93.1	Neutral	
Tiotropium bromide ^d^	Extracellular muscarinic receptor	−1.23 ^a^	−1.49 ^a^	n/a		59.1	Permanently cationic	
Ipratropium bromide ^d^	Extracellular muscarinic receptor	−1.20 ^a^	−1.75 ^a^	n/a		46.5	Permanently cationic	
Rifampicin ^c^	Intracellular bacteria	2.54 ^a^	0.3	1.7	6.70	217	Zwitterion	(29,30)
Ciprofloxacin ^c^	Intracellular bacteria	0.28	−1.11	6.30	8.60	74.6	Zwitterion	(24,26)

pK_a_a_: acidic pK_a_; pK_a_b_: basic pK_a_; Physicochemical data were expressed as experimental values where available except indicated: ^a^Predicted using ADMET Predictor^TM^ v7.0; ^b^Average value of two studies (27,28); Respiratory indications are ^c^ intracellular infections and ^d^COPD and asthma; ^e^Predicted LogP is expected to be higher than indicated, as the measured LogD_7.4_ is higher than this value

## Materials and Methods

### Chemicals and Reagents

1-Aminobenzotriazole, ammonium chloride, ciprofoxacin, clarithromycin, dimethyl sulphoxide, Imipramine, lactate dehydrogenase activity assay kit, monensin sodium salt, nigericin sodium salt, trypan blue 0.4% and verapamil hydrochloride were all from Sigma Aldrich Ltd., Dorset, UK. Budesonide, ipratropium bromide, fenoterol, formoterol, terbutaline and tiotropium bromide were all supplied by GlaxoSmithKline, UK. Chloroform and formaldehyde 37–41% were from Fisher Scientific, Loughborough, UK. Further chemicals include diazepam (Tocris Bioscience, Bristol, UK), methanol (VWR, UK), midazolam (Hoffman La Roche, Switzerland), Pierce BCA protein assay kit (Thermo Scientific, Loughborough, UK) and Lysotracker® Red DND-99 (Life Technologies, Paisley, UK). Reagents include bovine serum albumin and penicillin-streptomycin (Sigma Aldrich Ltd., Dorset, UK), collagen Type I rat tail (BD Biosciences, Oxford, UK), Dulbecco’s phosphate buffered saline and heat-inactivated foetal bovine serum (Life Techonolgies, Paisley, UK) and Kaighn’s modification of Ham’s F12 (Ham’s F12K) medium (American Tissue Culture Collection (ATCC), Mannasas, VA, USA).

### NR8383 Cell Culture and Maintenance

The NR8383 cell line (CRL-2192) was purchased from ATCC, Mannasas, VA, USA. Cells were grown in Ham’s F12K medium with 2 mM glutamine and 1.5 g/L sodium bicarbonate which was further supplemented with 15% heat-inactivated foetal bovine serum and 100 units/mL penicillin-100 μg/mL streptomycin to make complete growth medium (CGM). Cells, which were present as both floating and attached cell populations, were routinely maintained in tissue culture flasks. CGM was changed twice per week and involved removal of the floating cells from flasks, rapid addition of fresh CGM into the flasks to maintain adherent cells in medium and re-suspension of floating cells in CGM to be returned into flasks. The latter was performed following centrifugation of the cells at 142 *g* for 5 min (Eppendorf Centrifuge 5804, Cambridge, UK) at room temperature. Upon ~70% confluency was reached, cells were passaged by gentle scraping of the loosely attached cells into the existing culture medium. Following centrifugation at aforementioned conditions, cell pellet was gently re-suspended in 1 mL of CGM previously maintained at 37°C and seeded into new flasks at 2 × 10^5^ or 3.5 × 10^5^ cells per mL. Cell count and viability were assessed with trypan blue exclusion method using a haemocytometer under the light microscope (Olympus CK2, Olympus Optical Co., Japan). Cells were maintained by incubation at 37°C, 5% CO_2_ in a humidified atmosphere. Only cells with passage number up to 25 were used to ensure consistency in the cell line morphology.

### Drug Uptake in NR8383

The cellular uptake method was adapted from Ménochet *et al.* (2012) (31) and optimised for NR8383, as outlined herein. The studies were carried out in a 24-well plate format and the *in vitro* conditions were optimised in terms of cell detachment method from culture flasks, coating type of 24-well plates, seeding density, culturing time and drug incubation time points. Briefly, for cell detachment, gentle scraping *versus* Mg^2+^-Ca^2+^ free DPBS treatment of cells in culture flasks were tested. Considering the semi-adherent nature of AMs, cell seeding into non-coated, collagen-I and poly-D-lysine coated 24-well plates was investigated for cell attachment, distribution in the wells and detachment following DPBS washes during the uptake experiments. In addition, seeding densities of 0.36 and 0.72 × 10^6^ cells per well were assessed with respect to cell attachment, confluency following culture (plating efficiency) and potential cell detachment following DPBS washes. Culturing time ranging from 4 to 24 h was investigated for optimal cell attachment before carrying out uptake experiments. Uptake rate and K_p_ data were normalised by cell number determined indirectly by measuring the amount of protein in samples by bicinchoninic acid (BCA) protein assay (Thermo Scientific, Loughborough, UK). Following the optimisation, detachment of NR8383 from cell culture flasks was performed by gentle scraping prior to cell counting and seeding into collagen-I coated 24-well plates at 0.5 × 10^6^ cells per well. Cells were cultured for 16 h at 37°C, 5% CO_2_ (CO_2_ incubator, MCO-17AIC, Sanyo Biomedical, Loughborough, UK) before uptake studies performed over 1 to 10 min period. The details of method optimisation outlined above can be found in the Supplementary Material, Figure S2.

Initially, uptake experiments in NR8383 were carried out for all drugs at single substrate concentration of 5 μM at 37 and 4°C in order to delineate contribution of active uptake and passive diffusion processes. The drug uptake in NR8383 corresponding to these conditions was described by total uptake (CL_uptake_) and passive diffusion (CL_diff_) clearance terms, respectively. For a subset of selected drugs, accumulation was assessed over a range of drug concentrations (0.1–100 μM for imipramine, 1–100 μM for formoterol and clarithromycin) at 37°C. In addition, clarithromycin uptake kinetics was evaluated over this concentration range. Substrate solutions were prepared by dilution of the stock solutions in DPBS resulting in final DMSO content < 1%. Uptake studies were performed in the presence of 1 mM ABT, a nonspecific P450 inhibitor, in order to avoid any potential impact of phase I metabolism. Currently there is no evidence of the presence of any conjugation enzymes in this cell line. Following microscopy, the CGM was removed, cells were washed twice with 800 μL of either pre-warmed or ice-cold DPBS (for experiments at 37 and 4°C, respectively) and pre-incubation with 500 μL of pre-warmed or ice-cold ABT/DPBS was carried out for 20 min. Where ABT pre-treatment was not necessary, cell monolayers were treated with 500 μL of DPBS only. Once pre-incubation buffer was removed from the cell monolayers, incubations were started by addition of 400 μL of substrate solution (pre-warmed or ice-cold) in each well. Each substrate was incubated with the cells for 1, 2, 5 and 10 min and each time point at 37°C and 4°C. For 37°C experiments, incubation was carried out on a dry heater block maintained at 37°C throughout the experiment (Tecam Dri-block DB-3, Tecam, Princeton, NJ) and for 4°C experiments, the procedure was carried out on ice. Incubation with substrate was stopped by removal of the media with drug solution and washing the monolayers three times with 800 μL ice-cold DPBS. The back-diffusion of the drug from the cell into the medium was considered to be negligible under these conditions. The removed incubation media were retained for the measurement of drug medium concentrations and subsequent determination of K_p_ for each drug. The cells were lysed by addition of 200 μL ice-cold deionised water; cell lysates were kept at −20°C overnight and then processed further for LC-MS/MS analysis. Experiments were performed on three or more separate occasions for each drug investigated. Clarithromycin uptake at 5 μM was used as a control to assess the performance of individual experiment. The mean uptake of the 5 μM clarithromycin across a large number of experiments performed (*n* = 59) was 5.50 ± 2.96 μl/min/10^6^ cells. The amount of protein per well was measured using a BSA calibration curve and absorbances read at 562 nm (S22 Boeco UV-Visible Spectrophotometer, Hamburg, Germany). Determination of cell number from measured protein was reproducible across experiments (1 × 10^6^ cells = 0.25 ± 0.06 mg protein, *n* = 66).

### Assessment of Lysosomal Sequestration in NR8383 Cells with LysoTracker Red

Qualitative assessment of the localisation of lysosomes in NR8383 and lysosomal targeting of drugs investigated was made using LTR (Life Technologies, Paisley, UK). The protocol used for staining of the cells with LTR was adapted from a previous study (32). Clear flat bottom Ibidi 8-well chamber slides (Thistle Scientific, Glasgow, UK) were previously coated with 50 μg/mL collagen-I solution (4.05 mg/mL Collagen type I rat tail, BD Biosciences, Oxford, UK). NR8383 cells (250,000 cells/well) were plated in the slides and cultured for 16 h to 70–80% confluency and to ensure attachment. Stock solutions of NH_4_Cl, monensin and nigericin were prepared by diluting them in serum-free Ham’s F12K medium resulting in final incubation concentrations of 20 mM, 5 μM and 10 μM, respectively. In addition, stock solutions of clarithromycin, imipramine and formoterol were diluted in fresh serum-free Ham’s F12K medium containing 1 mM ABT to achieve final incubation concentrations of 5 μM. LysoTracker Red was added into the solutions of the chemical agents and the drugs to achieve a final concentration of 200 nM as used previously (14). A control solution of LTR at the same concentration was also prepared by dilution of the original DMSO stock in serum-free Ham’s F12K medium. Upon reaching confluency, cells were examined under the microscope (Olympus CK2, Olympus Optical Co., Japan), the CGM was removed and the cells were pre-treated with 300 μL warm DPBS containing 1 mM ABT for 20 min at 37°C in the presence of 5% CO_2_ (CO_2_ incubator, MCO-17AIC, Sanyo Biomedical, Loughborough, UK). Following this, the cells were treated with 300 μL of serum-free Ham’s F12K medium containing either LTR alone (control) or LTR with each of the chemical agents or selected drugs for 1 h at 37°C in the CO_2_ incubator and protected from light. Following the incubation, the cells were washed three times with 300 μL ice-cold DPBS and immediately fixed with 4% (*v/v*) formaldehyde in DPBS for 10 min at room temperature, protected from light. The cells were then treated with 50 mM NH_4_Cl in DPBS to quench the residual formaldehyde fluorescence for 5 min at room temperature. Three subsequent washes with ice-cold DPBS were performed before visualisation of LTR with a confocal laser scanning microscope (Zeiss LSM 510, Jena, Germany) using a C-Apochromat 40×/1.2 NA (Numerical Aperture) water-immersion objective and helium/neon laser (λ_excitation_ 543 nm, λ_emmision_ 560 nm). Images were collected and processed using Combi LSM-FCS v.3.2 and LSM Image Browser v4.2 software (Jena, Germany). Furthermore, Image J v1.47 software was used for the quantification of the fluorescence intensity of LTR in absence or presence of chemical agents and basic drugs (Image J, National Institutes for Health, US). The corrected total cell fluorescence (CTCF) was calculated as integrated density – (area of selected cell x mean fluorescence of background reading) (33). The CTCF of treated cells was compared to 100% control (LTR only).

### Lysosomal Sequestration Studies in NR8383

The extent of accumulation of drugs in lysosomes was assessed using the same uptake methodology, as described above in the absence and presence of NH_4_Cl and ionophores, monensin and nigericin. Preliminary investigation of imipramine and clarithromycin uptake was performed across a range of NH_4_Cl concentrations (10–50 mM), based on previous studies in the literature (12–14,34). In addition, a range of incubation conditions was explored, including pre-incubation and co-incubation alone and combined together. Once the conditions were optimised, further experiments were performed with 20 mM NH_4_Cl co-incubated with investigated drugs. The details of this optimisation work can be found in the Supplementary Material, Table S2 and Figure S3. Following the initial assessment with NH_4_Cl, confirmation of lysosomal sequestration for a number of selected drugs was further carried out by co-incubation of 5 μM monensin or 10 μM nigericin with the selected drugs. The concentrations of both ionophores used in this study were optimised based on previous reports (13,14,34) and in house solubility observations. Stock solutions of NH_4_Cl, monensin and nigericin were in deionised water, methanol and chloroform respectively. For incubations with NH_4_Cl, all drugs were prepared at 5 and 20 μM concentrations, with the exception of ciprofloxacin due to solubility issues at higher concentrations. In the case of clarithromycin, its accumulation was further evaluated in the presence and absence of NH_4_Cl over a wide substrate concentration range. The incubation time points with NH_4_Cl were up to 10 min based on the previous data on the changes in lysosomal pH with this agent reported in mouse peritoneal macrophages (35). For incubations with monensin and nigericin, all drugs were prepared at 5 μM concentration and the incubations were carried out for 5 min (single time point). Following the treatment of the cell monolayers with drug alone or drug with the agents mentioned, incubations were stopped by washing of the cells with ice-cold DPBS as described above, followed by cell lysis in deionised water. All incubations were carried out in duplicate and experiments were performed for three separate occasions. The removed incubation media were retained for the measurement of drug concentrations and subsequent determination of K_p_ for each compound. Analysis of samples in LC-MS/MS was performed after cells were kept at −20°C overnight.

### Cytotoxicity Assessment

Lactate dehydrogenase (LDH) assay kit (Sigma-Aldrich, Dorset, UK) was used to measure the cytotoxicity of the chemical agents used to assess the extent of lysosomal sequestration relative to untreated cells. In addition, the cytotoxicity was assessed at high drug concentrations (50 and 100 μM) in cases where uptake was investigated over a substrate concentration range. The viability of the untreated cells and the effect of the individual drugs and chemical agents, as well as the effect of their combined use were assessed. Therefore, four types of samples were included in the analysis; namely, the incubation medium consisting of DPBS alone (negative control), drug alone (*e.g.*, clarithromycin), chemical agents alone (*e.g.*, NH_4_Cl), and chemical agent and drug combination (*e.g.*, NH_4_Cl + clarithromycin). For the assessment of the individual chemical agents, same concentrations were used as in the lysosomal sequestration studies. The cytotoxicity in above samples was measured on three separate occasions. The LDH activity of the experimental samples was compared against the 100% activity of the LDH positive control included in the assay kit. All samples and standards were run in duplicate and absorbance values were measured using a plate reader (Tecan Safire, A-5082, Reading, UK). Data acquisition was performed using Magellan software (version 7.1, Tecan Group Ltd., Austria).

### Sample Preparation and LC-MS/MS Analysis

All cell lysates and media samples were thawed and quenched with methanol containing internal standard. The internal standard concentration was 1 μM for all drugs except for budesonide and rifampicin for which 0.1 μM was used. For cell lysate and media samples, equal volume of methanol/internal standard mixture (200 μL) was used. Media samples with substrate concentrations of 10 μM and above were diluted down to 2 μM with DPBS before quenching with the same volume of methanol/internal standard mixture (200 μL). This initial dilution was performed to avoid saturation of ionisation in LC-MS/MS at high concentrations. Samples were placed at −20°C for at least an hour to precipitate the proteins, followed by their centrifugation at 2500 rpm for 10 min (Eppendorf Centrifuge 5804, Cambridge, UK). Ten or 20 μL of supernatant was analysed by LC-MS/MS as detailed below.

Samples were analysed on a Waters Alliance 2795 HPLC system coupled to a Micromass Quattro Ultima mass spectrometer (Waters, Watford, UK) using electrospray positive ionisation mode. All compounds and their internal standards were separated on Luna C18 column (3 μm, 50 × 4.6 mm) (Phenomenex, Macclesfield, UK), except for fenoterol, terbutaline, ipratropium and tiotropium bromide which were separated on Luna Phenyl Hexyl column (3 μm, 50 × 4.6 mm) (Phenomenex, Macclesflield, UK). The mobile phases used were: Solvent A, 90% water, 10% methanol, and 0.05% formic acid; Solvent B, 10% water, 90% methanol, and 0.05% formic acid; Solvent C, 90% water, 10% methanol, and 1 mM ammonium acetate; and Solvent D, 10% water, 90% methanol, and 1 mM ammonium acetate. The gradient of mobile phases varied for each drug. The flow rate through HPLC was 1 mL/min, which was then split to 0.25 mL/min before entry into the mass spectrometer. For the Waters Ultima, the capillary voltage was 3.5 kV; desolvation and source temperatures were 350 and 125°C, respectively; desolvation gas and cone gas flow rates were 600 and 150 L/h, respectively. LC-MS/MS conditions for each individual drug and their corresponding internal standards are detailed in Supplementary Material, Table S3. A calibration standard, containing the compound of investigation at a concentration range covering that of the experimental samples with an additional zero blank, was prepared in the same matrix of the experimental samples and analysed twice (before and after the experimental samples) during each run, in order to confirm compound stability, consistent peak area for internal standard and any potential compound carry over. Samples were quantified against the standard curve using MassLynx v.4.1 (Waters Inc., Milford, MA). During analysis, only standards which were measured within 30% of the nominal concentration were included in the standard curve.

### Determination of Uptake Clearance Parameters

The CL_uptake_ and CL_diff_ were estimated at a single substrate concentration for each drug from the 37 and 4°C data, respectively. Uptake rate was determined from the slope of the linear regression of the cell concentration *vs.* time plot. Total uptake clearance was then estimated from the ratio of the uptake rate against the substrate concentration. Both CL_uptake_ and CL_diff_ were determined for each drug on a minimum three separate occasions. Uptake clearance attributed to the active process(es) was determined from the difference between CL_uptake_ and CL_diff_ for each drug (36).

### Determination of Drug Accumulation

Accumulation of drugs in NR8383 was determined by calculating K_p_ of all drugs at 5 μM at 10 min. K_p_ was determined by Eq. 1 as reported previously (36,37);

1 $$ {\mathrm{K}}_{\mathrm{p}}=\frac{{\mathrm{C}}_{\mathrm{cell}}}{{\mathrm{C}}_{\mathrm{medium}}} $$

where C_cell_ and C_medium_ represent concentration of the drug in the cell and medium, respectively. Both represent the measurements made at the final incubation time point (10 min). The K_p_ represents partition of the drug into total cell and reflects intracellular binding, subcellular distribution and active and passive uptake processes. C_cell_ was calculated by multiplying the measured lysate concentration with lysate volume (200 μL) and dividing this amount by the cell volume (1.04 μL/10^6^ cells, CV = 24%) and number of cells measured per well. Cell volume was calculated by multiplying the reported NR8383 volume of 4.2 μL/mg protein (38) with the amount of protein measured experimentally (0.25 ± 0.06 mg/10^6^ cells). While K_p_ was determined at a single substrate concentration for all the drugs investigated, the accumulation of 3 basic drugs including imipramine, clarithromycin and formoterol was also characterised over a concentration range at the same incubation time. The concentration dependence of drug accumulation was investigated by fitting the two-site binding model to the K_p_ and concentration data in GraFit v6.0.6 software (Erithacus Software Limited, Horley, UK). The model incorporates a saturable site and a linear function for unsaturated binding as shown in Eq. 2;

2 $$ {\mathrm{K}}_{\mathrm{p}}={\mathrm{K}}_{\mathrm{p},\mathrm{U}1, \max }-\frac{{\mathrm{K}}_{\mathrm{p},\mathrm{U}1, \max}\cdot {\mathrm{C}}_{\mathrm{medium}}}{{\mathrm{K}}_{\mathrm{U}1}+{\mathrm{C}}_{\mathrm{medium}}}+{\mathrm{K}}_{\mathrm{p}, \min } $$

where K_p,U1,max_ is maximum K_p_ for saturable uptake, K_p,min_ is K_p_ for nonsaturable uptake and K_U1_ is apparent saturable uptake equilibrium constant. K_p_ for maximum total uptake was calculated as the sum of K_p,U1,max_ and K_p,min_, as reported previously (39).

### Determination of Uptake Kinetic Parameters of Clarithromycin

The estimation of uptake kinetic parameters of clarithromycin was performed using the two-step method (31,36) for analysis in GraFit v6.0.6 (Erithacus Software Limited, Horley, UK). CL_diff_ was estimated from the slope of the linear regression of the substrate concentration *vs.* uptake rates estimated at 4°C and used as a constant parameter in Eq. 3. When estimating the uptake kinetic parameters in the presence of NH_4_Cl, CL_diff_ estimated at single substrate concentration was used a constant in the equation. Subsequently, kinetic parameters K_m_^′^ and V_max_ were obtained by nonlinear regression.

3 $$ \mathrm{v} = \frac{{\mathrm{V}}_{\max } \times {\mathrm{S}}_{\mathrm{m}\mathrm{ed}}\ }{{\mathrm{K}}_{\mathrm{m}}^{\prime } + {\mathrm{S}}_{\mathrm{m}\mathrm{ed}}} + {\mathrm{CL}}_{\mathrm{diff}} \times {\mathrm{S}}_{\mathrm{m}\mathrm{ed}} $$

where v is the uptake rate, K_m_^′^ is the binding affinity constant and V_max_ is the maximum uptake rate associated with an active process mediating uptake of clarothromycin into AM. S_med_ (μM) is the nominal media concentration corrected for fraction unbound in the media (fu_med_ = 0.93 ± 0.03). The latter was determined from the slope of the linear regression of the unbound concentration extrapolated at time 0 *vs.* the nominal incubation concentration plot.

### Determination of Lysosomal Sequestration

K_p_ and CL_uptake_ were determined as described above for each drug both under control condition and in the presence of a chemical agent affecting the pH gradient between cytosol and lysosomes. The reduction in K_p_ and CL_uptake_ of each drug in the presence of the chemical agents was expressed as a percentage relative to control and used as an indicator of the extent of lysosomal sequestration. A 50% reduction in both parameters was set as a cut-off for lysosomal accumulation, while also taking into account the physicochemical properties of the compounds (Table I) and the uncertainty associated with the experimental data.

The *in vitro* parameters were calculated on at least three separate occasions and the mean data were reported. When assessing the extent of lysosomal sequestration, the control and chemical agent treated cells were compared using the two-tailed, paired *t*-test in order to determine statistically significant difference between the two conditions. Values were reported as significant when *p* < 0.05.

## Results

### Drug Accumulation in NR8383 Cell Line

Accumulation of 10 drugs was assessed in NR8383 at a single substrate concentration (5 μM) at 37°C. Measured cell and media concentrations of all drugs were used to determine their K_p_, as shown in Table II. More than 500-fold range in K_p_ was observed in NR8383 for the current dataset. The most extensive accumulation in NR8383 was seen for imipramine and clarithromycin (K_p_ of 391 and 82, respectively). Ciprofloxacin, rifampicin, budesonide and formoterol accumulated in NR8383 to a similar extent (K_p_ values ranging between 25 and 43). For the remaining drugs, relatively less partitioning into cells was observed, as tiotropium, ipratropium bromide and fenoterol achieved a K_p_ between 5 and 9. In the case of terbutaline, the medium concentration of the compound remained higher than its cell concentration throughout the incubation period, resulting in K_p_ close to 1.

**Table II:** Drug Uptake Clearance and Cell-to-Medium Concentration Ratio (K_p_) of 10 Drugs in NR8383 at 5 μM Substrate Concentration. Data Represent Mean ± SD of at Least 3 Separate Experiments. CL_uptake_ and CL_diff_ are Total Uptake and Passive Diffusion Clearances, Respectively

Drug	CL_uptake_ (μL/min/10^6^ cells)	CL_diff_ (μL/min/10^6^ cells)	Contribution of passive (%)	K_p_
Imipramine	16.7 ± 4.3	5.06 ± 1.7	30.2	391 ± 108
Clarithromycin	6.0 ± 3.7	0.03 ± 0.03	0.53	81.6 ± 22.8
Formoterol	2.0 ± 0.7	0.08 ± 0.08	3.71	24.5 ± 8.71
Budesonide	1.5 ± 0.7	1.5 ± 0.9	97.2	37.6 ± 11.4
Rifampicin	1.4 ± 0.5	0.03 ± 0.01	1.87	38.6 ± 18.1
Ciprofloxacin	1.3 ± 0.4	0.3 ± 0.2	22.3	43.0 ± 21.1
Tiotropium bromide	0.4 ± 0.3	0.06 ± 0.04	16.3	8.74 ± 7.24
Fenoterol	0.3 ± 0.2	0.03 ± 0.01	8.03	5.69 ± 0.98
Ipratropium bromide	0.2 ± 0.2	0.01 ± 0.01	5.19	5.42 ± 3.38
Terbutaline	0.04 ± 0.01	0.02 ± 0.01	47.6	0.69 ± 0.16

**Fig. 1:**
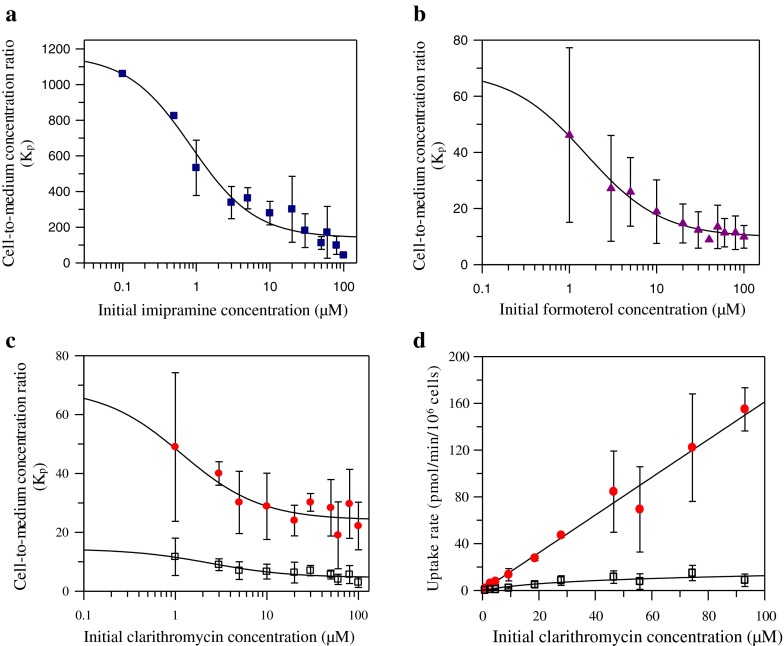
Cell-to-medium concentration ratio (K_p_) of imipramine (**a**), formoterol (**b**) and clarithromycin (**c**), and total uptake rate of clarithromycin (**d**) over a range of concentrations in NR8383 cells. Data represent mean ± SD of 3 experiments except for the first two data points (0.1–0.5 μM) of imipramine (*n* = 1). *Solid line* represents the fit for total uptake at 37°C. *Symbols* represent the observed data in the absence ( imipramine;  formoterol;  clarithromycin) and presence () of 20 mM NH_4_Cl at 37°C. For K_p_ and uptake kinetic parameter estimation, the fitting was obtained using the two-site binding model (Eq. 2) and the conventional two-step model (Eq. 3).

For a number of basic drugs, accumulation in the NR8383 was assessed further over a range of substrate concentrations. A concentration-dependent accumulation was observed for the drugs investigated, as illustrated in Fig. 1. The two-site binding model was fitted to the experimental data and the estimated uptake parameters K_p,U1,max_, K_p,min_ and K_U1_ are shown in Table III. In contrast to the K_p_ value obtained at 5 μM, imipramine maximum K_p_ of 1166 was estimated by the modelling approach (corresponds to concentration <0.1 μM). The K_p,min_ of 141, 24 and 10 was estimated for imipramine, clarithromycin and formoterol, respectively. The analysis indicated that half of maximal K_p_ associated with saturable uptake would be reached at concentrations below 2 μM for these three drugs (Table III). The fold difference between K_p,max_ and K_p,min_ ranged between 3 and 8 for clarithromycin and imipramine, respectively.

**Table III:** Uptake Parameter Values for 3 Drugs Investigated in NR8383 Over a Concentration Range. K_p,U1,max_, K_p,min_ and K_U1_ were Estimated Using the Two-Site Binding Model. Data Represent Mean ± SD of 3 Experiments

Drug	K_p,U1,max_	K_p,min_	K_U1_ (μM)	K_p,max_	K_p,max_/K_p,min_
Imipramine	1025 ± 99.6	141 ± 30.6	0.9 ± 0.3	1166	8.3
Clarithromycin	44.9 ± 16.6	24.2 ± 2.06	1.18 ± 1.45	69	2.9
Formoterol	59.2 ± 8.77	10.0 ± 0.92	1.54 ± 0.49	69	6.9

Uptake of 10 drugs was also assessed in NR8383 at 5 μM at 37 and 4°C in order to delineate contribution of active and passive diffusion processes to the overall uptake into AMs (Table II). The trends seen in the intracellular accumulation data were reflected in the uptake clearances, with over 400-fold range in CL_uptake_ in NR8383 observed for the current dataset (0.04–16.7 μL/min/10^6^ cells). Imipramine showed the highest uptake clearance in NR8383 (16.7 μL/min/10^6^ cells) with maximal contribution of the passive process to the total uptake of approximately 30%. Negligible CL_diff_ was observed for clarithromycin, suggesting a large contribution of an active process to its accumulation in NR8383. The same trend was observed for the majority of drugs with the exception of terbutaline and budesonide where contribution of passive diffusion to accumulation in NR8383 was >50% (Table II). Fenoterol, terbutaline, ipratropium and tiotropium bromide were marginally taken up into NR8383 cells with a CL_uptake_ of < 1 μL/min/10^6^ cells.

### Confocal Microscopic Examination of NR8383 Cells and Lysosomal Sequestration

**Fig. 2:**
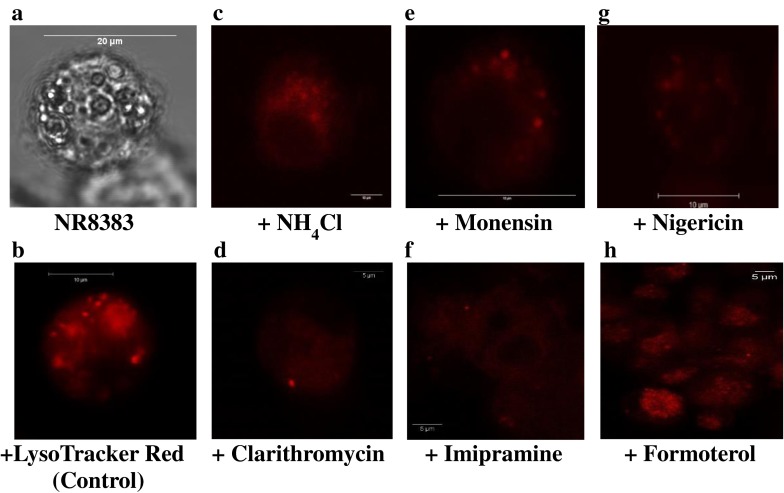
Qualitative assessment of lysosomal sequestration of LysoTracker Red (LTR) and three drugs studied in NR8383 by confocal microscopy: (**a**) differential interference contrast image of a single NR8383 cell treated with 200 nM LTR; (**b**) the same cell being excited to detect LTR localised in lysosomes under control conditions; the localisation of LTR in the lysosomes of NR8383 was reduced in presence of 20 mM NH4Cl (**c**), 5 μM monensin (**d**), 10 μM nigericin (**e**), 5 μM clarithromycin (**f**) and 5 μM imipramine (**g**), and showed minor changes in presence of 5 μM formoterol (**h**).

The imaging of NR8383 cells was performed in order to confirm localisation of functional lysosomes in NR8383 and assess the effect of the chemical agents on LTR accumulation in these organelles. In addition, the lysosomal targeting of selected drugs was investigated. A differential contrast image was taken every time prior to excitation of the cells for LTR detection. A representative of this image is shown in Fig. 2a, demonstrating the presence of cytoplasmic vesicular structures likely to represent acidic organelles including lysosomes. The corresponding fluorescent image of the same cell is illustrated in Fig. 2b where LTR was localised in lysosomes, confirming the presence of these organelles in NR8383 cells. Treatment of NR8383 with LTR in the presence of NH_4_Cl (20 mM), monensin (5 μM) and nigericin (10 μM) showed reduced fluorescent signal up to 85% for all agents. The reduced accumulation of LTR in the presence of these agents (Fig. 2c, e, g) confirmed lysosomal targeting of this basic probe in NR8383. Assuming the competition of basic drugs for lysosomal sequestration, LTR was co-incubated with 5 μM of clarithromycin, imipramine and formoterol. Clarithromycin and imipramine decreased LTR accumulation by 86 and 72%, respectively (Fig. 2d, f), confirming indirectly the lysosomotropic properties of these two drugs. In contrast, the localisation of LTR was still evident in the presence of formoterol (Fig. 2h) which caused only 25% reduction in LTR accumulation, suggesting its marginal targeting of NR8383 lysosomes.

### Cytotoxicity Assessment

Analysis of all three agents either on their own or when co-incubated with clarithromycin or imipramine showed minimal cytotoxicity (less than 2% compared to 100% positive control). At all times, the viability of the control (*i.e.*, untreated) cells was more than 95%. The measured cytotoxicity was less than 1% for imipramine at both 50 and 100 μM concentrations. For formoterol, the cytotoxicity was less than 5% compared to the positive control, whereas in the case of clarithromycin, the cytotoxicity levels were <3% and 10% for 50 μM and 100 μM concentrations, respectively.

### Assessment of Lysosomal Sequestration in NR8383

**Fig. 3:**
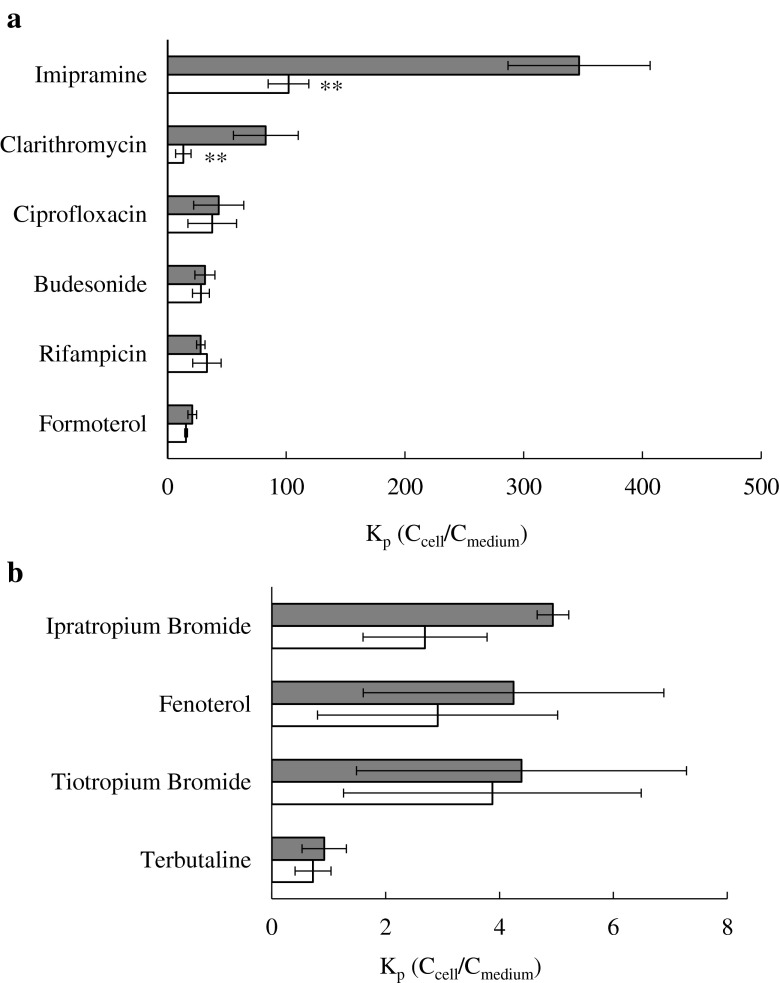
K_p_ (C_cell_/C_medium_) estimated in absence () (control) and presence () of 20 mM ammonium chloride (NH_4_Cl) at 5 μM concentration of (**a**) imipramine, clarithromycin, formoterol, rifampicin, budesonide, ciprofloxacin, and (**b**) ipratropium bromide, fenoterol, tiotropium bromide and terbutaline. Data represent mean ± SD of at least 3 experiments carried out at separate occasions (**, *p* < 0.01 by *t*-test).

Lysosomal sequestration of 10 drugs was assessed in NR8383 cells using three different chemical agents. The analysis was performed initially with 20 mM NH_4_Cl co-incubated with each drug at 37°C for 10 min. The pH of the incubation medium showed a minimal reduction in the presence of NH_4_Cl (<0.06 unit) relative to control conditions. The effect of NH_4_Cl on the intracellular accumulation of individual drugs is shown in Fig. 3. Of all the drugs investigated, the most pronounced reduction in intracellular accumulation was observed for clarithromycin, as K_p_ obtained at 5 μM decreased by 6-fold in the presence of NH_4_Cl relative to control condition, whereas in the case of imipramine 3.4-fold lower K_p_ was seen (Table IV). The presence of NH_4_Cl in the incubation did not significantly alter the intracellular accumulation of the remaining drugs (Table IV and Supplementary Material, Table S4). Even though K_p_ was reduced for some of the drugs (*e.g.*, by 46% for ipratropium bromide), the large uncertainty associated with the data (both control and + NH_4_Cl data) resulted in no significant difference between the two conditions. For the majority of drugs, the% reduction in K_p_ in the presence of NH_4_Cl was within 20% at both substrate concentrations.

**Table IV:** Cell-to-Medium Partition Coefficients (K_p_) Estimated Under Control and NH_4_Cl Treatment Conditions for 10 Drugs Investigated at 5 μM in NR8383. Percent Reduction in K_p_ in the Presence of NH_4_Cl (20 mM), monensin (5 μM) and nigericin (10 μM) is shown as an indicator of the extent of lysosomal Sequestration. Data Represent Mean ± SD of at Least 3 Experiments

				% Reduction in K_p_ by	
Drug	K_p_ Control	K_p_ + NH_4_Cl	NH_4_Cl	Monensin	Nigericin
Imipramine	347 ± 59.8	102 ± 17.1	71 ± 2.6^**^	75 ± 11^**^	66 ± 11^**^
Clarithromycin	82.7 ± 27.3	13.3 ± 6.50	84 ± 6.6^**^	77 ± 8.5^**^	82 ± 9.6^**^
Formoterol	20.8 ± 3.6	15.5 ± 1.16	24 ± 9.0	40 ± 7.5	27 ± 5.1^*^
Fenoterol	4.25 ± 2.64	2.91 ± 2.11	31 ± 23	27 ± 25	30 ± 12
Terbutaline	0.92 ± 0.39	0.72 ± 0.31	21 ± 5.0	n/a	n/a
Rifampicin	27.9 ± 3.54	33.2 ± 11.9	NR	n/a	n/a
Tiotropium bromide	4.39 ± 2.90	3.87 ± 2.61	12 ± 1.8	n/a	n/a
Ipratropium bromide	4.94 ± 0.30	2.69 ± 1.09	46 ± 21	n/a	n/a
Budesonide	31.5 ± 8.46	28.1 ± 7.12	10 ± 7.8	n/a	n/a
Ciprofloxacin	43.0 ± 21.1	37.6 ± 20.5	15 ± 13	n/a	n/a

*n/a* not available, *NR* no reduction in K_p_; ^*^, *p* < 0.05; ^**^, *p* < 0.01 by *t*-test

**Fig. 4:**
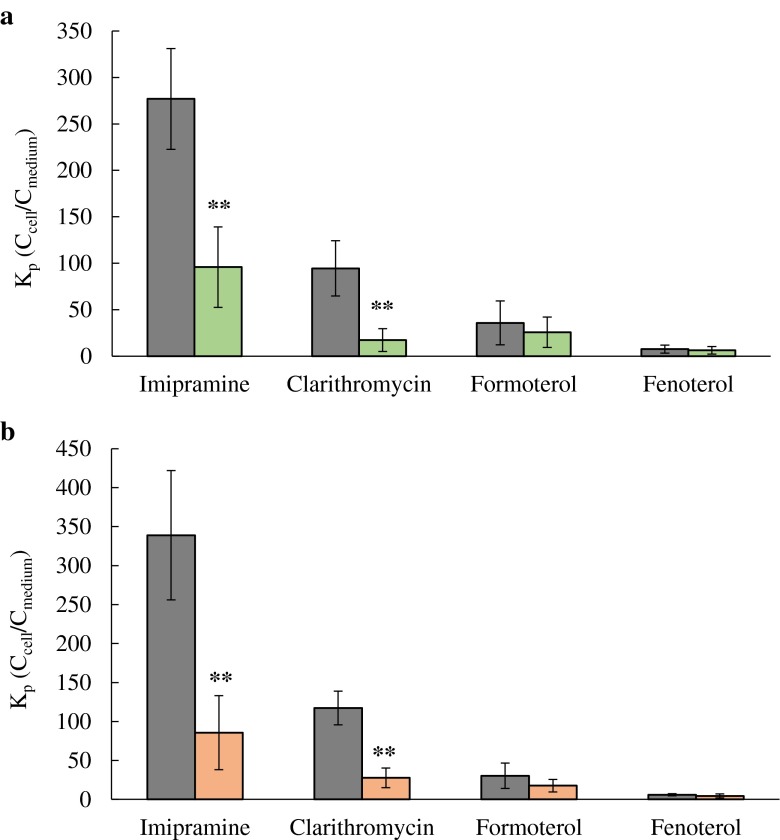
K_p_ (C_cell_/C_medium_) estimated in NR8383 in the absence () (control) and presence of (**a**) 10 μM nigericin () (**b**) 5 μM monensin () at 5 μM concentration of imipramine, clarithromycin, formoterol and fenoterol. Data represent mean ± SD of at least 3 experiments (**, *p* < 0.01 by *t*-test).

Lysosomal sequestration of imipramine, clarithromycin, formoterol and fenoterol was investigated further by monensin and nigericin; total K_p_ of these drugs in the absence and presence of these agents are shown in Fig. 4. The K_p_ of imipramine was significantly reduced in the presence of both monensin (by 66%) and nigericin (by 75%). The reduction of clarithromycin K_p_ was more pronounced in comparison to imipramine (77 and 82% by monensin and nigericin, respectively), in agreement with the effect of NH_4_Cl. In contrast, minor reduction in fenoterol and formoterol K_p_ in the presence of these agents was observed, corroborating findings from the studies with NH_4_Cl. The observations from confocal imaging studies confirmed the results outlined above, with regards to both the effectiveness of the chemical agents and the extent of lysosomal accumulation of clarithromycin, imipramine and formoterol (Fig. 2). In addition to K_p_, CL_uptake_ of each drug was estimated in the presence of these chemical agents; the effect of NH_4_Cl, monensin and nigericin on CL_uptake_ of the drugs investigated followed the same trends seen for K_p_ data (details are shown in the Supplementary Material, Table S5, Figures S4 and S5).

Among all drugs, the accumulation of clarithromycin in NR8383 in the presence of NH_4_Cl was further characterised over a range of clarithromycin concentrations (1–100 μM). Compared to control conditions, a marked reduction in clarithromycin K_p_ was observed at all drug concentrations (Fig. 1c). Estimated K_p,U1,max_ and K_p,min_ values in the presence of NH_4_Cl were 9.7 and 4.7, respectively, resulting in a mean maximum fitted K_p_ of 14.4. Assessment of uptake kinetics of clarithromycin under the control conditions showed nonsaturating conditions up to 100 μM and therefore, the kinetic parameters could not be determined. In contrast, in the presence of NH_4_Cl, the uptake rates of clarithromycin were reduced markedly at all substrate concentrations compared to the control condition (Fig. 1d) and showed a saturable uptake kinetic profile (despite highly variable rates at higher concentrations, CV > 30%). In the presence of NH_4_Cl, K_m_^′^ and V_max_ associated with the active uptake of clarithromycin into NR8383 were 28.5 μM and 12.4 pmol/min/10^6^ cells, respectively; the corresponding clearance was 0.43 μL/min/10^6^ cells. Considering estimated marginal contribution of the passive process, total clarithromycin CL_uptake_ (0.46 μL/min/10^6^ cells), was in agreement with the value obtained at a single concentration in the presence of NH_4_Cl (Supplementary Material, Figure S4).

## Discussion

Many clinically used drugs are basic and lipophilic, with a high propensity to accumulate in lysosome rich tissues, including the lungs. Depending on the location of intracellular drug targets the consequence of lysosomal sequestration varies, and could affect the efficacy of respiratory drugs targeting airway submucosal compartments (*e.g.*, β_2_-agonists and antimuscarinics). Drug accumulation in AMs may lead to formation of foamy macrophages (*e.g.*, in drug induced phospholipidosis) and may raise safety concerns. Currently, there is a paucity of *in vitro* studies investigating the accumulation of respiratory drugs and potential lysosomal distribution in either primary human AMs or macrophage cell lines. In the current study, comprehensive analysis of uptake and lysosomal sequestration of 10 drugs was performed in NR8383. This rat alveolar macrophage cell line was selected as a representative AM system due to its ease of culturing and reported comparable characteristics to freshly isolated rat AMs (17,18).

### Assessment of Drug Accumulation and Membrane Partitioning in NR8383

Following the optimisation of the methodology, the uptake of 10 drugs was investigated in NR8383 at a single concentration (5 μM) limited by the analytical quantification of the cellular concentrations of the drugs investigated. The analysis showed pronounced differences in the extent of accumulation illustrated by over a 400-fold range in both CL_uptake_ (0.04–17 μL/min/10^6^ cells) and K_p_ (0.7–391) for the drugs in the dataset. Imipramine and clarithromycin, as the two most lipophilic basic drugs, showed the highest intracellular accumulation in NR8383, in agreement with previous studies reporting accumulation of clarithromycin and a number of CADs in AMs *in vitro* (12,40–42). A number of studies also reported substantial imipramine accumulation in isolated perfused lungs of rats (estimated K_p_ of 17–44 depending on concentration), or other cell systems including rat hepatocytes (K_p,max_ 360) (37,43,44). Recently, clarithromycin K_p_ of ~16 was reported in NR8383; the estimate is based on measurements at 10 min at 37°C and is approximately 5-fold lower than the K_p_ obtained here (12). This discrepancy can be explained by the use of much higher initial clarithromycin concentration (50 μM) in the study by Togami *et al.* (2013) (12) compared to the current analysis. For the remaining respiratory drugs investigated, relatively lower intracellular accumulation in AMs was observed (Table II) reflective of their physicochemical properties. Most drugs have limited plasma membrane permeability due to being largely ionised and relatively hydrophilic at physiological pH, and are non-amphiphilic in structure, supporting no extensive partitioning into membranes (Table I).

In this present study, the mechanisms driving the uptake of respiratory drugs in AMs were investigated at both 37 and 4°C. Although the reliability of the 4°C approach to accurately determine passive diffusion is often questioned because of the reduced fluidity of the cellular membrane (45), this method was used as the presence of any membrane transporters in NR8383 is currently unknown, hence eliminating the possibility to assess the process by the use of specific transporter inhibitors. Uptake of most drugs into NR8383 was driven by an active process, reflected in <25% contribution of the passive diffusion to the total uptake for 7/10 drugs investigated. The largest contribution of active uptake process(es) to overall intracellular accumulation in NR8383 was observed for clarithromycin. This finding is in agreement with Togami *et al.* study (12) that showed a reduction in clarithromycin K_p_ by 90% in NR8383 at 4°C compared to its value at 37°C. In the case of rifampicin, formoterol, ipratropium and tiotropium bromide, the supporting evidence for their active uptake comes from other systems such as HEK293 cells, Xenopus laevis oocytes and human airway epithelial cells in which the involvement of OATP, OCT and OCTN transporters was highlighted (46–51).

In addition to the analysis at a single drug concentration, the accumulation of imipramine, clarithromycin and formoterol (all basic drugs) in NR8383 was characterised further by determining K_p_ over a range of substrate concentrations. The accumulation of these drugs was concentration-dependent and best described by a two-site model consisting of a saturable process, attributed to either transporter-mediated uptake and/or lysosomal sequestration, and a second linear process representing nonsaturable binding to membrane acidic phospholipids (22,37,39). The two-site binding model allowed determination of the maximal extent of accumulation (K_p,max_) of investigated drugs in AMs which can be difficult to assess analytically, as it requires use of very low substrate concentrations. The extent of saturable uptake was approximately 3-fold for clarithromycin, whereas the magnitude of this uptake was much greater for formoterol and imipramine (K_p,max_/K_p,min_ ratio of 6.9 and 8.3, respectively). The accumulation profile of imipramine in NR8383 is in agreement with the study by Hallifax and Houston (37) who also described its accumulation in rat hepatocytes as a two-site process including a high affinity-low capacity component (saturable) and a low affinity-high capacity process (nonsaturable). Similar concept was also previously proposed for imipramine accumulation in lysosomes isolated from rat liver (52), where the high affinity-low capacity site was attributed to lysosomes considering the effect of NH_4_Cl seen. Based on these, the K_p,min_ of the drugs at the highest substrate concentration can be assumed to represent membrane partitioning upon saturation of active processes. Imipramine, as a CAD well known to interact with membrane acidic phospholipids (53,54), showed the highest membrane partitioning (K_p,min_ of 141) whereas in the case of clarithromycin and formoterol K_p,min_ values were 5 to 14-fold lower (Table III). This less pronounced partitioning into membranes (lysosomal and cellular plasma membrane) is not surprising considering the non-amphiphilic and relatively less lipophilic nature of clarithromycin and formoterol relative to imipramine.

### Assessment of Lysosomal Sequestration of Drugs in NR8383

Lysosomal sequestration is an important active process that may contribute to intracellular drug accumulation. The qualitative assessment of this process was performed with LTR staining of NR8383 in the absence and presence of NH_4_Cl, monensin and nigericin, agents known to abolish lysosome-cytosol pH gradient. In the quantitative analysis, the reduction in total drug uptake clearance and accumulation by the mentioned chemical agents was assessed. This indirect method provides an advantage over methods requiring cell homogenisation and differential centrifugation to isolate lysosomes, as it is simple, rapid and allows a larger number of compounds to be assessed. In addition, it avoids issues of poor recovery, potential contamination of lysosomes and drug diffusion from these organelles during sample preparation. The extent of reduction in intracellular accumulation of investigated drugs in NR8383 obtained in the presence of chemical agents differed substantially for the drugs in the dataset. No or minimal reduction in K_p_ (<15%) was observed for zwitterions rifampicin and ciprofloxacin, neutral budesonide and permanently cationic tiotropium bromide. Among basic drugs, the reduction in K_p_ was in the range of 20–40% for formoterol, fenoterol and terbutaline. Out of all drugs, significant lysosomal accumulation in NR8383 was observed only for basic imipramine and clarithromycin (reduction in K_p_ of 65% and above). In the case of imipramine, the results were in agreement with the use of this drug as a prototypical CAD for the assessment of lysosomal sequestration, as demonstrated in numerous different *in vitro* systems either in the presence of the chemical agents (Supplementary Material, Table S1) or LTR (14,55). Although clarithromycin is cationic, it is not an amphiphilic drug due to the absence of the typical hydrophobic moiety (an aromatic and/or aliphatic ring structure) present in many CADs (56). However, macrolides have been indicated to be one of the few exceptions which lack the typical CAD structure yet cause phospholipidosis linked to lysosomal sequestration (5). Lysosomal accumulation of clarithromycin was observed for the first time across a concentration range, supported by the reduction in its K_p_ in the presence of NH_4_Cl at all drug concentrations including 100 μM. These findings suggest that even at high concentrations, its intracellular accumulation was not driven solely by membrane partitioning but also by the lysosome-cytosol pH gradient. Therefore, the true membrane partitioning (*i.e.*, K_p,min_) of clarithromycin is likely to be closer to the estimate obtained in the presence of NH_4_Cl. No such data currently exist in the literature to allow direct comparison. Togami and colleagues previously showed that treatment of NR8383 with NH_4_Cl reduced the amount of clarithromycin in the cells by approximately half and the fraction of the drug in the granules compartment (containing the lysosomes) by 75% (12), in excellent agreement with the reduction in K_p_ obtained in the current study.

The inclusion of basic drugs in this study was driven by their basic pK_a_ (range from 8.14 to 9.9), which together with the lysosome-cytosol pH gradient suggested potential accumulation in the lysosomes (predicted K_p,lysosome_ based on pH partitioning ranged between 315 and 443 for terbutaline and imipramine, respectively). However, under the experimental conditions used herein, 3 out of 5 basic drugs with LogP < 3 failed to show substantial lysosomal accumulation, suggesting that both sufficient lipophilicity and ionisation are required for lysosomal accumulation within relatively short incubation times. This finding was in agreement with a number of studies reporting lysosomal sequestration for drugs with LogP > 2 and pK_a_ between 6.5 and 11 (14,55). However, it is also evident that the mentioned physicochemical space is not definitive and that a number of drugs with these properties may fail to accumulate in the lysosomes. The discrepancy may be explained by the previous reports showing that drugs with similar octanol-water partition coefficients in unionised and ionised forms did not accumulate in the lysosomes to the same extent (mitochondrial accumulation was observed instead). This finding was in contrast to drugs for which partition coefficients of the ionised form were much lower relative to neutral (all have the above mentioned physicochemical properties) (15).

An important finding of the current lysosomal sequestration studies was the altered accumulation kinetics of clarithromycin in NR8383 when its lysosomal sequestration was significantly reduced in the presence of NH_4_Cl. While it was not possible to determine the uptake kinetic parameters for clarithromycin under control incubation conditions, the initial uptake rates were substantially reduced in the presence of NH_4_Cl and the kinetic parameter estimates could be obtained. It is uncertain whether these estimates truly reflect the existence of a membrane transporter in NR8383 and further investigation of transporters expressed in this cell line is required. The existing evidence for clarithromycin being a substrate for membrane transporters is conflicting. Although it was previously shown that it was subject to active hepatic uptake (36), a more recent study demonstrated that clarithromycin was not a substrate for OATPs and OCT1 *in vitro* (transfected HEK293 cells) and *in vivo* in OATP and OAT gene knockout mice (57). One potential explanation for the more saturable kinetics under NH_4_Cl condition is the saturation of lysosomal accumulation with increasing clarithromycin concentration. These results further suggest that for CADs, macrolides and other basic lipophilic drugs which accumulate in the lysosomes, the uptake kinetic parameter estimates obtained may not represent the true transporter affinities and uptake rates without adequate consideration of the lysosomal sequestration. This is particularly important if *in vitro* generated uptake kinetic parameters are to be used in physiologically-based pharmacokinetic models to predict *in vivo* transporter-mediated disposition of these drugs (58–61). Considering many lysosomotropic drugs are cationic and amphiphilic, these results warrant the need for studies to investigate the impact of lysosomal sequestration on uptake kinetics of known OCT and OCTN substrates.

In conclusion, the current study is the first to provide a comprehensive analysis of the extent of accumulation of a range of respiratory drugs in alveolar macrophages and the mechanisms contributing to the observed intracellular drug concentrations. The current data suggest minimal sequestration of inhaled drugs in lysosomes of alveolar macrophages; however, formulation and particle size also need to be taken into consideration in the translation of the findings to the *in vivo* setting. The study has highlighted the importance of both lysosomal sequestration and binding to acidic phospholipids for extensive cellular accumulation in AMs, which are distinct to transporter-mediated cellular uptake. Clarithromycin example clearly emphasizes the relevance of lysosomal sequestration on the understanding of the cellular accumulation kinetics and interplay with other active processes affecting intracellular drug concentrations. The abundance of lysosomes in NR8383, together with the data presented here, indicate potential application of this cell line in preclinical drug development as a routine system for investigation of lysosomal sequestration of new drug candidates. In addition, NR8383 may be used to predict the extent of respiratory drug accumulation in human AMs and to validate mechanistic *in silico* models developed to predict subcellular drug distribution. Therefore, this study provides a considerable scope for future research in the area of the prediction of respiratory drug accumulation in human alveolar macrophages.

## Electronic supplementary material

ESM 1 (DOCX 473 kb)

## Abbreviations

ABT: 1-aminobenzotriazole
AM(s): Alveolar macrophage(s)
BSA: Bovine serum albumin
CAD: Cationic amphiphilic drug
CGM: Complete growth medium
CL_diff_: Passive diffusion clearance
CL_uptake_: Total uptake clearance
DPBS: Dulbecco’s phosphate buffered saline
K_p_: Cell-to-medium concentration ratio
LC-MS/MS: Liquid chromatography with tandem mass spectrometry
LTR: LysoTracker Red
NH_4_Cl: Ammonium chloride
