# Supplementary material for: In Vitro Assessment of Uptake and Lysosomal Sequestration of Respiratory Drugs in Alveolar Macrophage Cell Line NR8383
Source: Pharm Res. 2015 Jul 30;32(12):3937–51. doi: 10.1007/s11095-015-1753-8 (PMC4628094; doi:10.1007/s11095-015-1753-8)
Supplement: Supplementary file 1 — (DOCX 473 kb) [file 11095_2015_1753_MOESM1_ESM.docx]

**Supplementary Data for the article PHAM-D-15-00169**

***In vitro assessment of uptake and lysosomal sequestration of respiratory drugs in alveolar macrophage cell line NR8383***

Ayşe Ufuk, Graham Somers, J. Brian Houston and Aleksandra Galetin


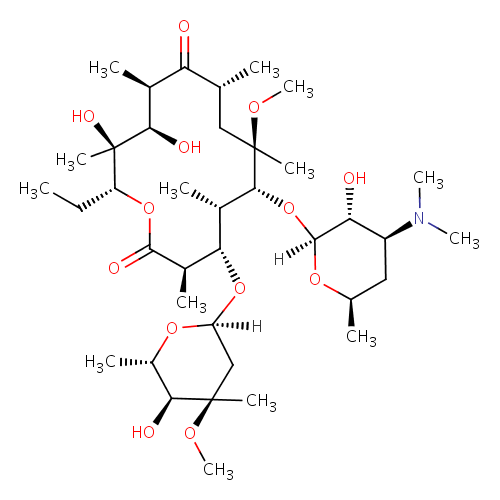

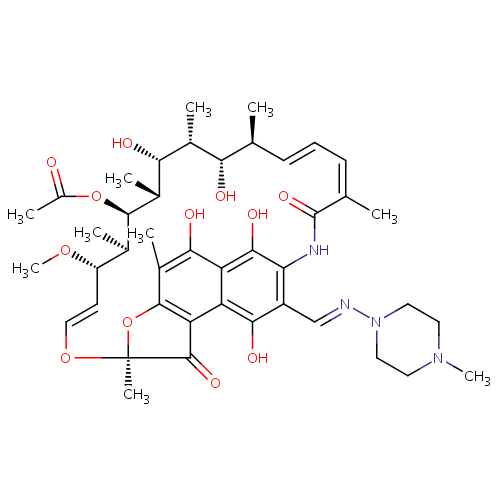

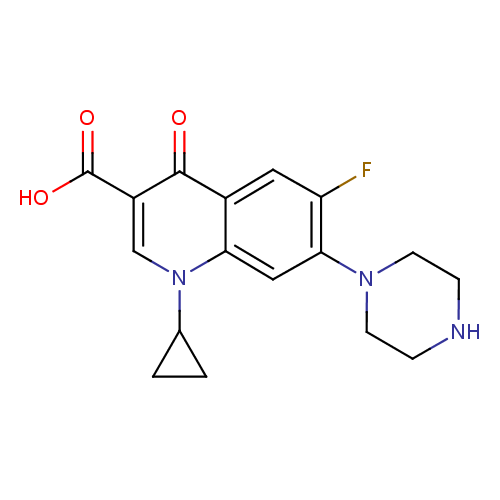

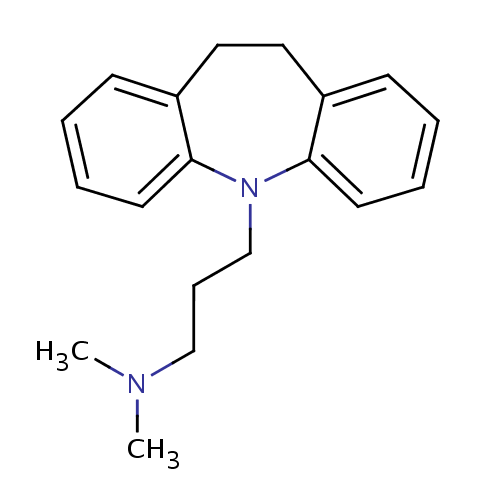

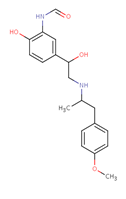


Clarithromycin Rifampicin Ciprofloxacin Imipramine Formoterol


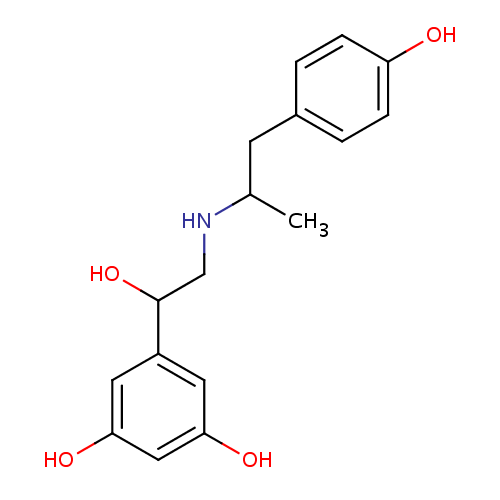

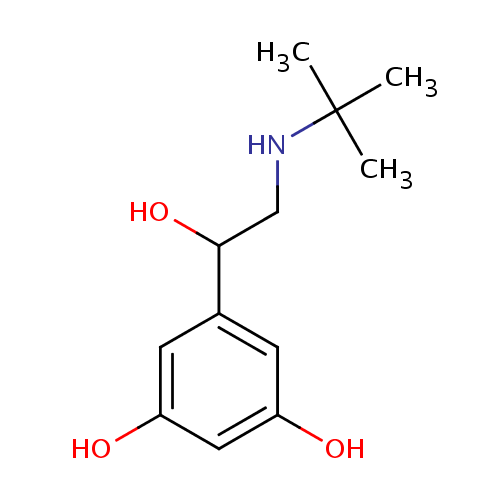

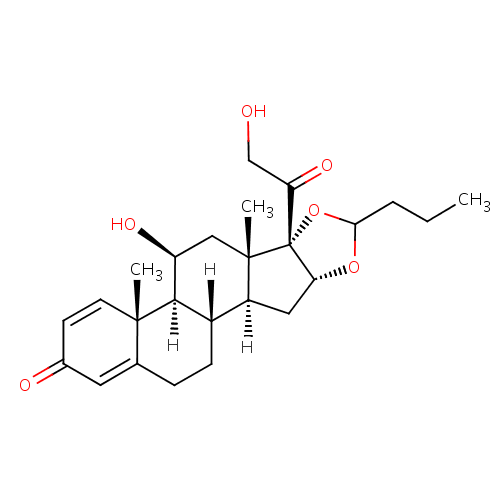

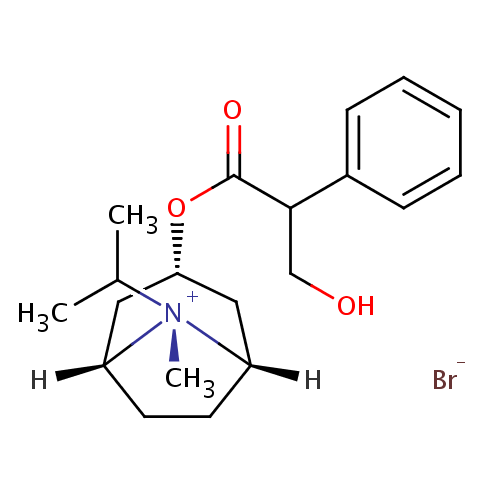

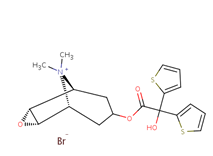


Fenoterol Terbutaline Budesonide Ipratropium Bromide Tiotropium Bromide

**Figure S1** Chemical structures of drugs investigated in NR8383


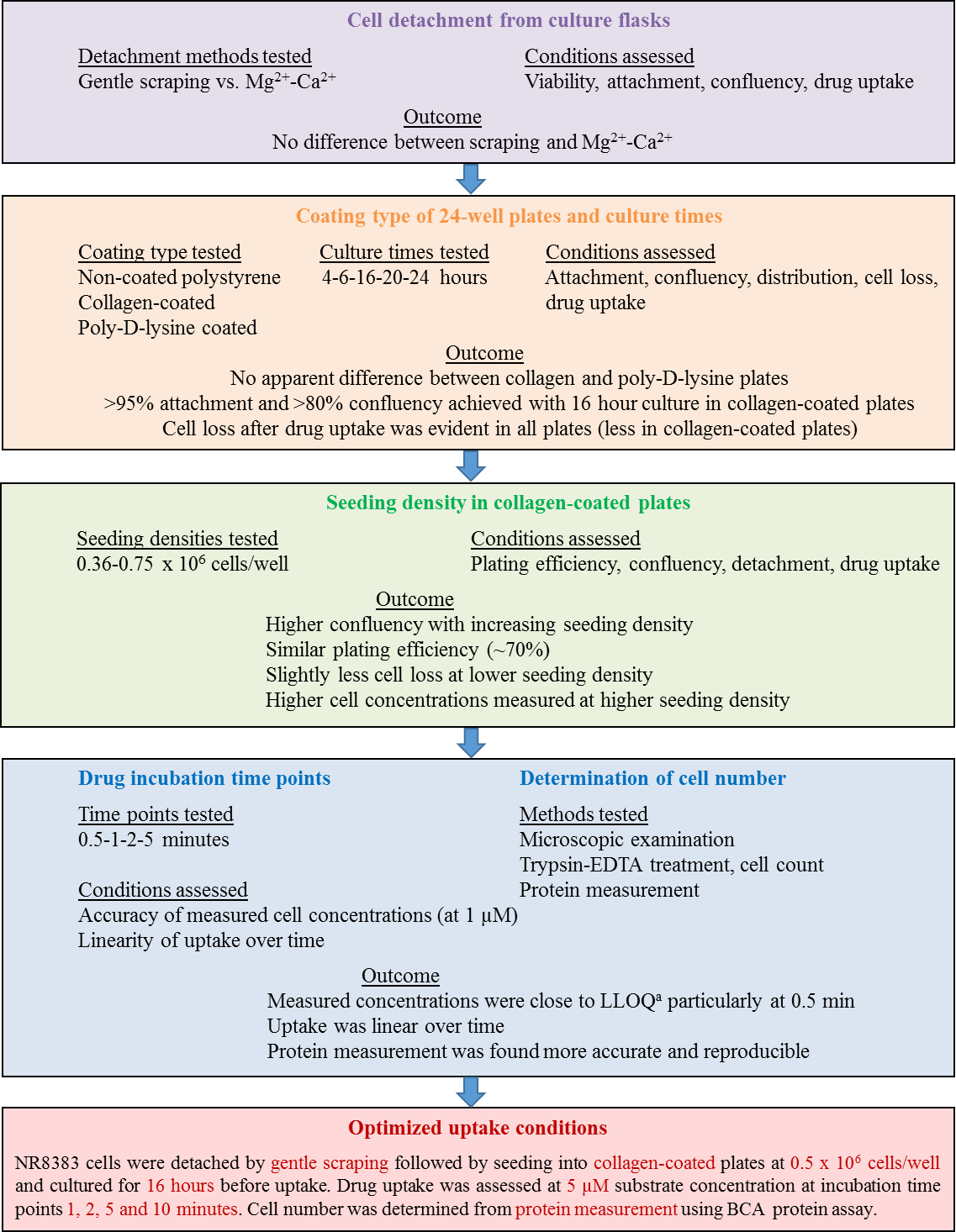
^a^ LLOQ: lower limit of quantification

**Figure S2** Schematic of optimisation of drug uptake conditions for NR8383 cells

**Figure S3** Total uptake clearance (CL_uptake_) of 5 and 20 µM imipramine and clarithromycin in NR8383 in the absence (■) and presence of 20 mM NH_4_Cl under different incubation conditions. (■) co-incubation; (■) pre-incubation; (■) pre- and co-incubation. Data are from a single experiment

**a**

******

*****

**b**

**Figure S4** CL_uptake_ estimated in absence (■) (control) and presence (□) of 20 mM ammonium chloride (NH_4_Cl) at 5 µM concentration of (a) imipramine, clarithromycin, formoterol, rifampicin, budesonide, ciprofloxacin, and (b) ipratropium bromide, fenoterol, tiotropium bromide and terbutaline. Data represent mean ± SD of at least 3 experiments carried out at separate occasions (*, *p* < 0.05; **, *p* < 0.01 by t-test)

**a**

*****

******

**b**

******

*****

**Figure S5** CL_uptake_ estimated in NR8383 in the absence (■) (control) and presence of (a) 10 µM nigericin (■) (b) 5 µM monensin (■) at 5 µM concentration of imipramine, clarithromycin, formoterol and fenoterol. Data represent mean ± SD of at least 3 experiments (*, p < 0.05; **, p < 0.01 by t-test)

**Table S1** Collation of literature studies investigating subcellular distribution of drugs in different in vitro systems using ammonium chloride (NH_4_Cl), monensin and nigericin

| **References** | **[NH_4_Cl] (mM)** | ***In vitro* system** | **Drugs** | **Concentration (µM)** | **pK_a_** | **LogP** | **Reduction in total uptake (%)** | **Time (min)** |
| --- | --- | --- | --- | --- | --- | --- | --- | --- |
| **(**[**1**](#_ENREF_1)**)** | 10 | Mouse peritoneal macrophages | Methylamine | 50 | 10.6 | -0.57 | 54 | 120 |
| **(**[**2**](#_ENREF_2)**)** | 10 | Hepatocytes isolated from male Wistar rats | Chloroquine | 10  100 | 8.4, 10.8 | 5.82 | 93  77 | 15 |
| **(**[**3**](#_ENREF_3)**)** | 20 | Tissue slices from male Sprague-Dawley rats | Desipramine  Chloroquine  Diazepam  Thiopental | 5  10  5  10 | 10.2  8.1, 10.1  3.5  7.5 | 4.53  5.82  2.9  2.78^a^ | Lung: 23^b^; Liver: 15  Lung: 66^b^; Liver: 53^b^  Lung: 2.6  NR | 60 |
| **(**[**4**](#_ENREF_4)**)** | 50 | Lysosomes isolated from male Wistar rat liver | Imipramine | 10^-4^ | 9.5 | 4.8 | 70^b^ | 10 |
| **(**[**5**](#_ENREF_5)**)** | 20 | Tissue slices from male Wistar rats | Promazine  Imipramine  Amitriptyline  Fluoxetine  Sertraline  Carbamazepine | 5  5  5  5  5  5 | Values ranging from 9.4-9.5 | 4.0  4.6  4.9  4.7  5.45  2.3 | Lung: 46.8^b^; Liver: 59^b^  Lung: 59^b^; Liver: 58^b^  Lung: 63^b^; Liver: 40^b^  Lung: 21^b^; Liver: 1.5  Lung: 42^b^; Liver: 20^b^  Lung: 5.4; Liver: 16.1 | 60 |
| **(**[**6**](#_ENREF_6)**)** | 10 | Lung granule fraction containing lysosomes from male Wistar rats | Chlorpromazine  Promethazine  Imipramine  Trihexylphenidyl  Biperiden  Pentazocine  Chloroquine | 1  1  1  1  1  1  1 | 9.3  9.1  9.5  8.7  8.8  8.5  8.4, 10.8 | 5.19  4.81  4.77  4.49  4.25  3.31  5.82 | 8  17  31  54  56  77  87 | 10 |
| **(**[**7**](#_ENREF_7)**)** | 3 mg/min/kg (i.v. infusion) | Tissue slices from male Wistar rats | Biperiden  Trihexyphenidyl | 3.2 mg/kg  (i.v. injection) | 8.8  8.7 | 4.25  4.49 | Lung: 77^b^; Liver: 2.5  Lung: 68^b^; Liver: 11 | 360 |

**^a^** Predicted by MarvinSketch v6.2.1 (ChemAxon Ltd.); ^b^ Reported statistically significant difference between control and treatment conditions; NR: no reduction

**Table S1 continued**

| **References** | **[NH_4_Cl] (mM)** | ***In vitro* system** | **Drugs** | **Concentration (µM)** | **pK_a_** | **LogP** | **Reduction in total uptake (%)** | **Time (min)** |
| --- | --- | --- | --- | --- | --- | --- | --- | --- |
| **(**[**8**](#_ENREF_8)**)** | 20 | Tissue slices from male Wistar rats | Thioridazine  Imipramine  Amitriptyline  Fluoxetine  Sertraline | 5  5  5  5  5 | Values ranging from 9.4-9.5 | Values ranging from  4.6-5.45 | Lung: 41^b^; Liver: 75^b^  Lung: 35^b^; Liver: 57^b^  Lung: 56^b^; Liver: 68^b^  Lung: 41^b^; Liver: 52^b^  Lung: 40^b^; Liver: 42^b^ | 60 |
| **(**[**9**](#_ENREF_9)**)** | 20 | Tissue slices from male Wistar rats | Perazine  Imipramine  Amitriptyline  Fluoxetine  Sertraline | 5  5  5  5  5 | 3.21, 8.14  Other values ranging from  9.4-9.5 | 2.95  4.6  4.9  4.7  5.45 | Lung: 58^b^; Liver: 61^b^  Lung: 49; Liver: 62^b^  Lung: 47^b^; Liver: 67^b^  Lung: 39^b^; Liver: 50^b^  Lung: 45^b^; Liver: 29^b^ | 60 |
| **(**[**10**](#_ENREF_10)**)** | 20 | Brain slices from male Wistar rats | Promazine  Thioridazine  Perazine  Imipramine  Amitriptyline  Fluoxetine  Sertraline | 5  5  5  5  5  5  5 | Values ranging from  3.2-9.5 | 4.0  5.13  2.95  4.6  4.9  4.7  5.45 | Brain: 20  Brain: 62^b^  Brain: 55^b^  Brain: 41^b^  Brain: 32^b^  Brain: 16.5  Brain: 47^b^ | 60 |
| **(**[**11**](#_ENREF_11)**)** | 10 | NR8383 cell line | Telithromycin | 50 | 2.4, 5.0, 8.7 | 5.3^a^ | 80^b^ | 120 |
| **(**[**12**](#_ENREF_12)**)** | 10 | NR8383 cell line and granule fraction (containing lysosomes) | Azithromycin  Clarithromycin | 50  50 | 8.1, 8.8  9.0 | 4.02  3.15 | Granule: 97^c^  Granule: 72^bd^  Granule: 88^c^  Granule: 48^bd^ | 120  30  60  30 |
| **(**[**13**](#_ENREF_13)**)** | 50 | Cell line of immortalized hepatocytes (Fa2N-4) | Propranolol  Imipramine | 1  1 | 9.67  9.20 | 2.58  4.28 | 52  60  48  47 | 5  30  5  30 |

^a^ Predicted by MarvinSketch v6.2.1 (ChemAxon Ltd.); ^b^ Reported statistically significant difference between control and treatment conditions; ^c^ before and ^d^ after subcellular fractionation

**Table S1 continued**

| **References** | **[Monensin] (µM)** | ***In vitro* system** | **Drugs** | **Concentration (µM)** | **pK_a_** | **LogP** | **Reduction of total uptake (%)** | **Time**  **(min)** |
| --- | --- | --- | --- | --- | --- | --- | --- | --- |
| **(**[**3**](#_ENREF_3)**)** | 10 | Tissue slices from male Sprague-Dawley rats | Desipramine  Chloroquine  Diazepam  Thiopental | 5  10  5  10 | 10.2  8.1, 10.1  3.5  7.5 | 4.53  5.82  2.9  2.78^a^ | Lung: 12^b^; Liver: NR  Lung: 73^b^ ; Liver: 44  Lung: 1.8  Lung: 10 | 60 |
| **(**[**14**](#_ENREF_14)**)** | 10 | Tissue slices from male Wistar rats | Promazine  Imipramine  Amitriptyline  Fluoxetine  Sertraline  Carbamazepine | 5  5  5  5  5  5 | Values ranging from 9.4-9.5 | 4.0  4.6  4.9  4.7  5.5  2.3 | Lung: 71^b^; Liver: 86^b^  Lung: 64^b^; Liver: 48^b^  Lung: 70^b^; Liver: 48^b^  Lung: 23^b^; Liver: 26^b^  Lung: 50^b^; Liver: 19^b^  Lung: 5.4; Liver: 0 | 60 |
| **(**[**8**](#_ENREF_8)**)** | 10 | Tissue slices from male Wistar rats | Thioridazine | 5 | 9.5 | 5.3 | Lung: 35^b^; Liver: 82^b^ | 60 |
| **(**[**9**](#_ENREF_9)**)** | 10 | Tissue slices from male Wistar rats | Perazine | 5 | 3.21, 8.14 | 2.95 | Lung: 71.6^b^; Liver: 65^b^ | 60 |
| **(**[**10**](#_ENREF_10)**)** | 10 | Brain slices from male Wistar rats | Promazine  Thioridazine  Perazine  Imipramine  Amitriptyline  Fluoxetine  Sertraline | 5  5  5  5  5  5  5 | Values ranging from  3.2-9.5 | Values ranging from 2.95-5.45 | Brain: 35  Brain: 58^b^  Brain: 55^b^  Brain: 37^b^  Brain: 31^b^  Brain: 22  Brain: 59^b^ | 60 |
| **(**[**15**](#_ENREF_15)**)** | 0.26  10 | Primary rat cerebellar neurons | Imipramine | 10^-4^ | 9.5 | 4.8 | 50  83 | 30 |

^a^ Predicted by MarvinSketch v6.2.1 (ChemAxon Ltd.); ^b^ Reported statistically significant difference between control and treatment conditions; NR: no reduction

**Table S1 continued**

| **References** | **[Nigericin] (µM)** | ***In vitro* system** | **Drugs** | **Concentration (µM)** | **pK_a_** | **LogP** | **Reduction of total uptake (%)** | **Time**  **(min)** |
| --- | --- | --- | --- | --- | --- | --- | --- | --- |
| **(**[**4**](#_ENREF_4)**)** | 50 | Lysosomes isolated from male Wistar rat liver | Imipramine | 0.001 | 9.5 | 4.8 | 70^b^ | 10 |
| **(**[**1**](#_ENREF_1)**)** | 5 µg/ml  (~7 µM) | Mouse peritoneal macrophages | Methylamine | 50 | 10.6 | -0.57 | 49 | 120 |
| **(**[**16**](#_ENREF_16)**)** | 2.5 | Lysosomes isolated from Male Wistar rat liver | Imipramine | 1 | 9.5 | 4.8 | 93  (relative to control without ATP)  99  (relative to control with ATP) | 20 |
|  |  |  |  |  |  |  |  |  |
| **References** | **[Nigericin**  **+Monensin] (µM)** | ***In vitro* system** | **Drugs** | **Concentration (µM)** | **pK_a_** | **LogP** | **Reduction of total uptake (%)** | **Time (min)** |
| **(**[**13**](#_ENREF_13)**)** | 10 µM +  20 µM | Cell line of immortalized hepatocytes (Fa2N-4) | Propranolol  Imipramine | 1  1 | 9.67  9.20 | 2.58  4.28 | 67  46 | 5 |

References for experimental drug physicochemical data: Desipramine ([17](#_ENREF_17)); Diazepam, biperiden and trihexyphenidyl ([5](#_ENREF_5)); Azithromycin ([18](#_ENREF_18)); Methylamine ([19](#_ENREF_19))

**Table S2** Percent reduction in uptake clearance of 5 and 20 µM imipramine and clarithromycin in the presence of 10, 20 and 50 mM NH_4_Cl

| **Drug** |  | **Reduction in uptake clearance (%)** | |
| --- | --- | --- | --- |
|  |  | **5 µM** | **20 µM** |
|  | + 10 mM NH_4_Cl | 58 | 71 |
| Imipramine | + 20 mM NH_4_Cl | 72 | 77 |
|  | + 50 mM NH_4_Cl | 80 | 78 |
|  | + 10 mM NH_4_Cl | 85 | 85 |
| Clarithromycin | + 20 mM NH_4_Cl | 93 | 89 |
|  | + 50 mM NH_4_Cl | 94 | 93 |

**Table S3** Summary of LC-MS/MS conditions used for assessing uptake and lysosomal trapping of 10 drugs in NR8383 cell line

| **Compound** | **Mass transition (m/z)** | **Cone voltage (V)** | **Collision voltage (eV)** | **Retention time**  **(min)** | **LLOQ (µM)** | **Internal Standard** | **Mass transition (m/z)** | **Cone voltage (V)** | **Collision voltage (eV)** | **Retention time (min)** |
| --- | --- | --- | --- | --- | --- | --- | --- | --- | --- | --- |
| Clarithromycin | 748.4>158.2 | 70 | 31 | 2.7 | 0.001 | Midazolam | 326.0>291.2 | 70 | 25 | 3.0 |
| Imipramine | 281.3>86.2 | 60 | 21 | 2.4 | 0.002 | Diazepam | 285.1>257.1 | 60 | 21 | 2.87 |
| Rifampicin | 821.5>789.3 | 50 | 15 | 2.7 | 0.010 | Verapamil | 455.3>165.1 | 60 | 30 | 2.5 |
| Ciprofloxacin | 332.1>314.3 | 80 | 20 | 2.3 | 0.010 | Midazolam | 326.0>291.2 | 70 | 25 | 2.7 |
| Formoterol | 345.3>149.1 | 25 | 20 | 2.35 | 0.002 | Midazolam | 326.0>291.2 | 70 | 25 | 2.8 |
| Fenoterol | 304.2>107.1 | 50 | 30 | 2.56 | 0.002 | Midazolam | 326.0>291.2 | 70 | 25 | 3.13 |
| Terbutaline | 226.3>152.2 | 60 | 15 | 2.8 | 0.002 | Midazolam | 326.0>291.2 | 70 | 25 | 3.0 |
| Budesonide | 431.35>413.35 | 70 | 8 | 3.48 | 0.010 | Verapamil | 455.3>165.1 | 60 | 30 | 2.95 |
| Ipratropium bromide | 332.35>166.2 | 85 | 25 | 2.8 | 0.002 | Midazolam | 326.0>291.2 | 70 | 25 | 3.1 |
| Tiotropium bromide | 392.3>152.15 | 75 | 25 | 2.9 | 0.002 | Midazolam | 326.0>291.2 | 70 | 25 | 3.12 |

LLOQ: lower limit of quantification

**Table S4** Cell-to-medium partition coefficients (K_p_) estimated under control and NH_4_Cl treatment conditions for 9 drugs investigated at 20 µM in NR8383. Percent reduction in K_p_ in the presence of NH_4_Cl (20 mM) is shown as an indicator of the extent of lysosomal sequestration. Data represent mean ± SD of at least 3 experiments

| **Drug** | **K_p_ Control** | **K_p_ + NH_4_Cl** | **% Reduction in K_p_ by NH_4_Cl** |
| --- | --- | --- | --- |
| Imipramine | 237 ± 29.3 | 81.3 ± 6.50 | 66 ± 2.4^**^ |
| Clarithromycin | 58.2 ± 11.6 | 14.2 ± 4.44 | 75 ± 8.3^*^ |
| Formoterol | 26.5 ± 6.54 | 19.2 ± 4.13 | 27 ± 8.1^*^ |
| Fenoterol | 1.50 ± 0.93 | 0.96 ± 0.33 | 27 ± 24 |
| Terbutaline | 0.79 ± 0.46 | 0.56 ± 0.29 | 22 ^a^ |
| Rifampicin | 34.6 ± 12.1 | 33.3 ± 12.2 | 12 ^a^ |
| Tiotropium bromide | 2.40 ± 1.00 | 2.00 ± 0.64 | 35 ± 26 |
| Ipratropium bromide | 3.71 ± 2.54 | 2.16 ± 1.37 | 39 ± 9.1 |
| Budesonide | 34.0 ± 12.5 | 31.0 ± 12.0 | 6.6 ± 4.1 |

^a^ Data represent mean of 2 experiments therefore standard deviation was not included; n/a, not available; NR, no reduction in K_p_; ^*^, *p* < 0.05; ^**^, *p* < 0.01 by t-test

**Table S5** Estimated reduction of total uptake clearance (CL_uptake_) of investigated drugs by NH_4_Cl, monensin and nigericin. Lysosomal sequestration was assessed at 5 and 20 µM drug concentration using NH_4_Cl (20 mM) and at 5 µM only using monensin (5 µM) and nigericin (10 µM). Data represent mean ± SD of at least 3 experiments

|  | % Reduction in CL_uptake_ by | | | |
| --- | --- | --- | --- | --- |
|  | **NH_4_Cl**  **(20 mM)** | | **Monensin**  **(5 µM)** | **Nigericin**  **(10 µM)** |
| Drugs | **At 5 µM** | **At 20 µM** | **At 5 µM** | **At 5 µM** |
| Imipramine | 68 ± 6.1^*^ | 59 ± 12^*^ | 72 ± 10^*^ | 62 ± 12^*^ |
| Clarithromycin | 85 ± 7.5^*^ | 78 ± 9.0^*^ | 78 ± 8.0^**^ | 84 ± 9.2^**^ |
| Formoterol | 25 ± 14 | 27 ± 13 | 41 ± 5.6 | 19 ± 12 |
| Fenoterol | 36 ± 12 | 22 ± 28 | 29 ± 21 | 25 ± 15 |
| Terbutaline | 15 ± 15 | 36 ± 23 | n/a | n/a |
| Budesonide | 18 ± 15 | NR | n/a | n/a |
| Ciprofloxacin | NR | n/a | n/a | n/a |
| Rifampicin | NR | NR | n/a | n/a |
| Ipratropium bromide | 45 ± 21 | 33 ± 16 | n/a | n/a |
| Tiotropium bromide | 15 ^a^ | 28 ^a^ | n/a | n/a |

^a^ Data represent mean of 2 experiments therefore standard deviation was not included; n/a, not available; NR, no reduction in CL_uptake_; ^*^, *p* < 0.05; ^**^, *p* < 0.01 by t-test

**References for Table S1**

1. Ohkuma S, Poole B. Cytoplasmic vacuolation of mouse peritoneal macrophages and the uptake into lysosomes of weakly basic substances. J Cell Biol. 1981;90(3):656-64.

2. MacIntyre AC, Cutler DJ. Role of lysosomes in hepatic accumulation of chloroquine. J Pharm Sci. 1988;77(3):196-9.

3. Daniel WA, Bickel MH, Honegger UE. The contribution of lysosomal trapping in the uptake of desipramine and chloroquine by different tissues. Pharmacol Toxicol. 1995;77(6):402-6.

4. Ishizaki J, Yokogawa K, Hirano M, Nakashima E, Sai Y, Ohkuma S, et al. Contribution of lysosomes to the subcellular distribution of basic drugs in the rat liver. Pharm Res. 1996;13(6):902-6.

5. Ishizaki J, Yokogawa K, Nakashima E, Ichimura F. Relationships between the hepatic intrinsic clearance or blood cell-plasma partition coefficient in the rabbit and the lipophilicity of basic drugs. J Pharm Pharmacol. 1997;49(8):768-72.

6. Ishizaki J, Yokogawa K, Nakashima E, Ohkuma S, Ichimura F. Uptake of basic drugs into rat lung granule fraction in vitro. Biol Pharm Bull. 1998;21(8):858-61.

7. Ishizaki J, Yokogawa K, Nakashima E, Ohkuma S, Ichimura F. Influence of ammonium chloride on the tissue distribution of anticholinergic drugs in rats. J Pharm Pharmacol. 1998;50(7):761-6.

8. Daniel WA, Wojcikowski J. The role of lysosomes in the cellular distribution of thioridazine and potential drug interactions. Toxicol Appl Pharmacol. 1999;158(2):115-24.

9. Daniel WA, Wojcikowski J. Lysosomal trapping as an important mechanism involved in the cellular distribution of perazine and in pharmacokinetic interaction with antidepressants. Eur Neuropsychopharmacol. 1999;9(6):483-91.

10. Daniel WA, Wojcikowski J, Palucha A. Intracellular distribution of psychotropic drugs in the grey and white matter of the brain: the role of lysosomal trapping. Br J Pharmacol. 2001;134(4):807-14.

11. Togami K, Chono S, Seki T, Morimoto K. Intracellular pharmacokinetics of telithromycin, a ketolide antibiotic, in alveolar macrophages. J Pharm Pharmacol. 2010;62(1):71-5.

12. Togami K, Chono S, Morimoto K. Subcellular Distribution of Azithromycin and Clarithromycin in Rat Alveolar Macrophages (NR8383) in Vitro. Biol Pharm Bull. 2013;36(9):1494-9.

13. Kazmi F, Hensley T, Pope C, Funk RS, Loewen GJ, Buckley DB, et al. Lysosomal sequestration (trapping) of lipophilic amine (cationic amphiphilic) drugs in immortalized human hepatocytes (Fa2N-4 cells). Drug Metab Dispos. 2013;41(4):897-905.

14. Daniel WA, Wojcikowski J. Contribution of lysosomal trapping to the total tissue uptake of psychotropic drugs. Pharmacol Toxicol. 1997;80(2):62-8.

15. Novelli A, Lysko PG, Henneberry RC. Uptake of imipramine in neurons cultured from rat cerebellum. Brain Res. 1987;411(2):291-7.

16. Ishizaki J, Yokogawa K, Ichimura F, Ohkuma S. Uptake of imipramine in rat liver lysosomes in vitro and its inhibition by basic drugs. J Pharmacol Exp Ther. 2000;294(3):1088-98.

17. Zhao Y, Jona J, Chow DT, Rong H, Semin D, Xia X, et al. High-throughput logP measurement using parallel liquid chromatography/ultraviolet/mass spectrometry and sample-pooling. Rapid Commun Mass Spectrom. 2002;16(16):1548-55.

18. McFarland JW, Berger CM, Froshauer SA, Hayashi SF, Hecker SJ, Jaynes BH, et al. Quantitative structure-activity relationships among macrolide antibacterial agents: in vitro and in vivo potency against Pasteurella multocida. J Med Chem. 1997;40(9):1340-6.

19. Dunn WJ, Nagy PI. Relative log P and solution structure for small organic solutes in the chloroform/water system using monte carlo methods. J Comput Chem. 1992;13(4):468-77.
